# Extinction magnitude of animals in the near future

**DOI:** 10.1038/s41598-022-23369-5

**Published:** 2022-11-23

**Authors:** Kunio Kaiho

**Affiliations:** Department of Earth Science, Tohoku University, Aoba-Aza, Aramaki, Aoba-Ku, Sendai, 980-8578 Japan

**Keywords:** Climate sciences, Ecology, Environmental sciences

## Abstract

There have been five major mass extinctions and some minor mass extinctions of animals since early animal diversification 540–520 Myr ago. It is said that a sixth mass extinction is already underway. However, the future extinction magnitude has not been quantitatively estimated. Here, I show that the sixth major mass extinction (defined as > 60% species loss) will be avoided, but a minor mass extinction, 20–50% animal species loss (1% now), will occur when humans cause nuclear war and/or fail to stop increasing greenhouse gas (GHG) emissions, pollution, and deforestation until 2060–2080 CE. When humans decrease GHG emissions, pollution, and deforestation in 40 years and prevent nuclear war in the future, 10–15% animal species loss will occur. Humans should stop not only industrial GHG emissions but also deforestation, environmental pollution, and nuclear war to prevent this mass extinction. When humans fail to stop these processes, significant decreases in biodiversity and the human population and a collapse of ecological balance will occur on Earth.

## Introduction

The ongoing species extinction rates, which reached the 1% level on land and the 0% level in seas from 1800–1900 to 2010^1–3^, are far from the major mass extinction magnitude (> 60%)^4^; however, the percentage of threatened species reaches 28% of all species at present on Earth^5^. Recently, some papers have suggested that a major mass extinction will occur due to human activity in the near future^1–3^. Since five major mass extinctions have occurred since animals became common on Earth 540–520 Myr ago^4,6^, this expected major mass extinction is called the sixth major mass extinction^1–3^. However, analyses of future extinction magnitudes have not yet been reported due to unknown relationships between the causes of future extinctions and the different causes of the Phanerozoic mass extinctions and the current extinction^4^.

The causes of mass extinctions in the Phanerozoic after the invasion of land by plants and animals are summarized as climate change, pollution, deforestation, and sunlight reduction. Changes in these four categories should have always occurred throughout the major mass extinctions, as evidenced by many geochemical analyses^4,7^. The ongoing current-future extinction of animals occurred in parallel with rapid global warming, with deforestation and pollution marked by mercury, CO, and black carbon emissions and plastic production^3^. Only the sunlight reduction category is not ongoing. When a nuclear war occurs, stratospheric soot is formed, thus causing sunlight reductions and global cooling^15^. Therefore, when nuclear war occurs, all four causes are active.

**Figure 1:**
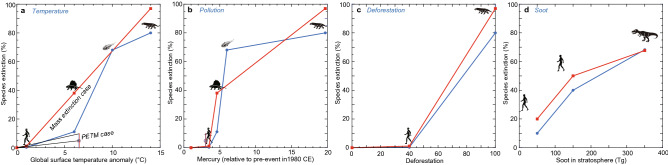
Relationships between the four causes of mass extinctions and the species extinction magnitude. The causes include global surface temperature anomalies^4^ (**a**), mercury amounts (relative to pre-event values) as a proxy for pollution (**b**)^10,22,29–31^, the deforestation area % (**c**)^12,18,19,24^, and the amount of soot present in the stratosphere inducing sunlight reduction and global cooling due to nuclear war in future or the asteroid impact at the K–Pg boundary (**d**)^14^ (see Methods and Supplementary Table S1). Data in **a**–**c** are from the end-Ordovician, end-Guadalupian, end-Permian, and Holocene–Anthropocene, characterized by global warming and terrestrial–nearshore animal extinctions. Blue circle: marine animals. Red square: terrestrial tetrapods. Open blue circles and red squares show data at the Paleocene–Eocene Thermal Maximum (PETM), which lacks deforestation, stratospheric aerosol formation causing global cooling and a mass extinction. There are the mass extinction case and PETM case on global surface temperature anomaly (**a**). An increase in global surface temperature by 6.5 °C, which is converted from the sea surface temperature (SST) anomaly (4–5 °C) of Zachos et al*.*^17^ using the figure of Kaiho^4^ for the conversion) at the Paleocene–Eocene thermal maximum (PETM). These data are shown in Supplementary Table S1.

**Figure 2:**
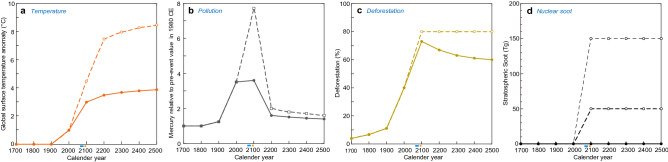
Changes in global surface temperature anomalies (**a**)^8,9^, mercury concentrations (relative to pre-event values) as a proxy for pollution (see Methods and Supplementary Table S2 for future mercury rate) (**b**)^11,25,26^, deforestation areas % (see Methods and Supplementary Table S2 for future deforestation) (**c**)^12,28^, and stratospheric soot amounts introduced by nuclear war inducing sunlight reduction and global cooling (**d**)^15^ from CE 1700 to 2500. Solid line in (**a**–**c**): the most likely case. Dushed line in (**a**–**c**): the worst case (see “Methods”). (**d**) shows stratospheric soot amount by two nuclear war scenarios after Coupe et al.^15^ and no nuclear war case. The light blue and orange bars under the horizontal axes show the timing of the stoppage of GHG emissions in the most likely case and in the worst case, respectively. These data are shown in Supplementary Table S2.

**Figure 3:**
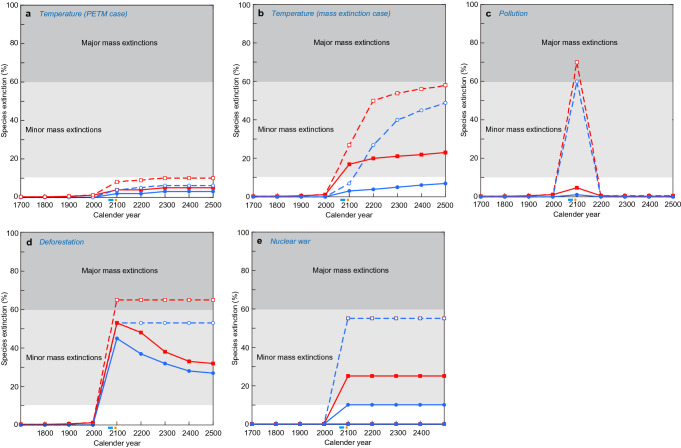
Animal species extinction percentages in the marine (blue) and terrestrial (red) realms from CE 1700 to 2500 resulting from global surface temperature changes in the PETM case (**a**), those in the mass extinction case (**b**), pollution (**c**), deforestation (**d**), and stratospheric soot introduced by nuclear war (**e**) in the most likely case (solid line) and the worst case (dushed line). This figure is made from data in Figs. 1 and 2. The light blue and orange bars under the horizontal axes show the timing of the stoppage of GHG emissions in the most likely case and in the worst case, respectively. These data are shown in Supplementary Tables S3 and S4.

**Figure 4:**
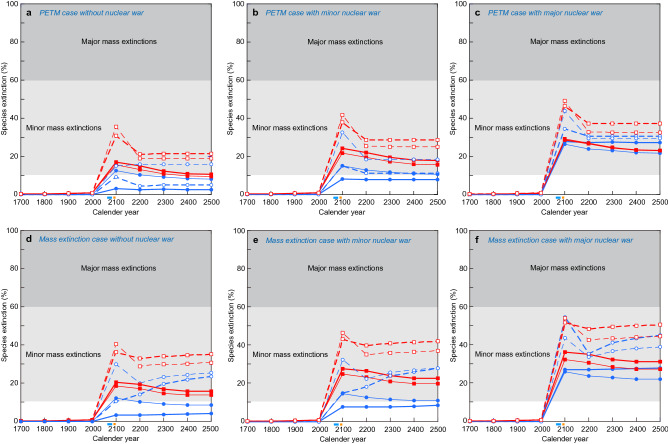
Animal species extinction percentages in the marine (blue circle) and terrestrial (red square) realms from CE 1700 to 2500 by all causes without nuclear war in the PETM case (**a**), those under minor nuclear war in the PETM case (**b**), those under major nuclear war in the PETM case (**c**), those without nuclear war in the mass extinction case (**d**), those under minor nuclear war in the mass extinction case (**e**), those under major nuclear war in the mass extinction case (**f**), in the most likely case (solid lines with closed dots) and the worst case (dushed lines with open dots). Thick lines: different contribution case. Thin lines: equal contribution case. The light blue and orange bars under the horizontal axes show the timing of the stoppage of GHG emissions in the most likely case and in the worst case, respectively. The maximum values of species extinction (%) correspond to the final species extinction magnitude. These data are shown in Supplementary Tables S5–S8. The maximum extinction magnitude occurred in ~ 2100 CE under no nuclear war case.

Although the relatively large surface temperature anomalies correlate well with the large extinction magnitudes of marine animals and terrestrial tetrapods in the Phanerozoic^4^, no relationships between other causes and extinction magnitudes in the Phanerozoic have been reported. First, I clarify the relevant relationships (Fig. 1). Second, I estimate the changes in the four causes listed above from 1700 to 2500 CE (Fig. 2). Third, I approximate the future extinction magnitude monitored by each cause and all causes together (Figs. 3, 4). The four causes are monitored in both the past and future by (i) measured historical global surface temperature anomalies^4^ and estimated future anomalies^8,9^, (ii) the increased rate of mercury concentration in sediments, used herein as a representative proxy for past pollution^10^ and CO_2_ emission amounts accompanied by pollutant materials for future pollution^11^, (iii) occupancy percentages of deforestation areas in pre-event forest areas for the past^12^ and human population harmonized with deforestation for the future^13^, and (iv) the amounts of stratospheric soot resulting from the K–Pg asteroid impact for the past^14^ and estimated nuclear war^15^ for the future. Only soot introduced by nuclear war is independent of the other causes, whereas the other causes are related both in the past and future. I analyze (i) the former three causes of global warming events on land and shallow seas because the current ongoing crisis is accompanied by global warming and (ii) the global cooling event at the Cretaceous–Paleogene boundary (K–Pg) for the latter cause, soot, because the formation of aerosols causing reduced sunlight is a common cause of major and minor mass extinctions^4,14,16^. The rapid global warming itself may not cause a mass extinction, because the Paleocene–Eocene thermal maximum (PETM) at 56 Myr ago characterized by global warming^17^ lacks deforestation^18,19^ and a mass extinction^20,21^. The ongoing biotic crisis without nuclear war is similar to PETM in having global warming, mercury concentration^22^, and lacking stratospheric aerosols causing global cooling. Whereas, the ongoing crisis with nuclear war will be similar to the past mass extinctions, whereas the ongoing crisis without nuclear war is similar to PETM except for deforestation. Therefore, I calculated animal species extinction magnitude (%) under the PETM case and the mass extinction case.

## Results

### The relationship between each cause and the species extinction magnitude

The first analysis indicates the following four results on the relationship between each cause and the species extinction magnitude. There is a positive relationship between global surface temperature anomalies and the species extinction magnitude under the mass extinction case^4^ (Fig. 1a). Under the PETM case, species extinction magnitude could have been < 10% because of no mass extinction. The larger mercury amount ratio values measured during extinction events compared to the pre-event values correspond to the relatively high extinction percentages in the marine and terrestrial realms (Fig. 1b). Both marine and terrestrial extinction magnitudes were low (1%)^1,2^ under at least < 40% deforestation compared to forests in ~ 4000 BC^12^; however, the 100% deforestation rate identified from plant fossil data in the Permian–Triassic transition^23,24^ corresponded to 80% and 97% species extinctions in sea and land regions, respectively (Figs. 1c). Globally distributed stratospheric soot absorbing sunlight and inducing global cooling can be a cause of animal extinctions^14^. The stratospheric soot amounts measured in correspondence with the K–Pg asteroid impact event and future estimated nuclear wars^15^ and the resulting global cooling anomalies are predicted to induce 10–20%, 40–50%, and 70% species extinctions when minor nuclear war (e.g., between India and Pakistan), major nuclear war (e.g., between USA and Russia), and the K–Pg impact event occur, respectively (Fig. 1d).

### Changes in the proxies representing the four causes

The second analysis indicates the following results regarding the changes in the proxies representing the four causes. In the most likely case, global surface temperature anomalies will reach 3.0 °C in 2100 CE, 3.5 °C in 2200 CE, and 3.8 °C in 2500 CE^8^ on average with global warming from 1861 to 1880 CE (Fig. 2a). A large increase in pollution is predicted from 1800 to 2100 CE, followed by a decrease until 2500 CE (Fig. 2b)^11,25,26^. Ongoing deforestation will likely mostly stop increasing in 2100 CE and then slightly decrease between 2200 and 2500 CE in the most likely case as the human population slightly decreases^27^; in the worst case, ongoing deforestation will continue at the maximum level because humans will not return farmland or cities to wild forests (Fig. 2c). Figure 2d shows the increased stratospheric soot amount resulting from nuclear war at three levels, namely, no nuclear war, minor nuclear war (e.g., between India and Pakistan), and major nuclear war (e.g., between the USA and Russia)^15^. The introduction of stratospheric soot by nuclear war will cause sunlight reduction, causing global cooling over a few years.

### Species extinction magnitude changes

The third analysis indicates the following results on the species extinction magnitude changes resulting from each cause using Figs. 1 and 2 and Supplementary Tables S1 and S2 (Fig. 3, Supplementary Tables S3, S4) and all causes using Fig. 3 and Supplementary Table S3, S4 (Fig. 4, Supplementary Tables S5–S8). The extinction magnitudes shown in Fig. 3 correspond to the case in which the extinction magnitudes controlled by the four causes can be monitored using only a single cause. The species extinction magnitude resulting from temperature anomalies will increase from 2000 to 2500 CE, and be low in PETM case (Fig. 3a) and higher in the mass extinction case (Fig. 3b). The species extinction magnitude estimated by pollution will peak at approximately 2100 CE and then decrease rapidly to an extinction rate of zero (Fig. 3c). The extinction rate induced by deforestation will peak in approximately 2100 CE, then gradually decrease to 60% of the maximum magnitude or maintain the maximum value (Fig. 3d). The extinction severity caused by stratospheric soot will depend on the occurrence magnitude of nuclear war calculated from the number of weapons deployed, and the average explosive power of the nuclear weapons^15^ (Fig. 3e shows the occurrences in each century).

Under no nuclear war case, the species extinction magnitudes, in percentages, obtained in correspondence with the four causes under two different contribution scenarios in the PETM case indicate that 3–13% marine animal species loss and 15–17% terrestrial tetrapod species loss will occur under the most likely case, and 9–16% marine animal species loss and 31–36% terrestrial tetrapod species loss will occur under the worst case (Fig. 4a, Supplementary Table S9). Those in mass extinction case indicate that 4–12% marine animal species loss and 19–21% terrestrial tetrapod species loss will occur under the most likely case, 24–30% marine animal species loss and 36–41% terrestrial tetrapod species loss will occur under the worst case (Fig. 4d, Supplementary Table S9).

Those in minor nuclear war case in the PETM case indicate that 8–15% marine animal species loss and 22–24% terrestrial tetrapod species loss will occur under the most likely case, 15–33% marine animal species loss and 38–42% terrestrial tetrapod species loss will occur under the worst case (Fig. 4b, Supplementary Table S9). Those in mass extinction case show that 9–15% marine animal species loss and 25–28% terrestrial tetrapod species loss will occur under the most likely case, and 28–33% marine animal species loss and 43–47% terrestrial tetrapod species loss will occur under the worst case (Fig. 4e, Supplementary Table S9).

Those in major nuclear war case in the PETM case indicate that 26–28% marine animal species loss and 29% terrestrial tetrapod species loss will occur under the most likely case, 34–44% marine animal species loss and 46–49% terrestrial tetrapod species loss will occur under the worst case (Fig. 4c, Supplementary Table S9). Those in mass extinction case show that 26–28% marine animal species loss and 32–36% terrestrial tetrapod species loss will occur under the most likely case, 44–55% marine animal species loss and 52–54% terrestrial tetrapod species loss will occur under the worst case (Fig. 4c, Supplementary Table S9).

## Discussion

**Figure 5:**
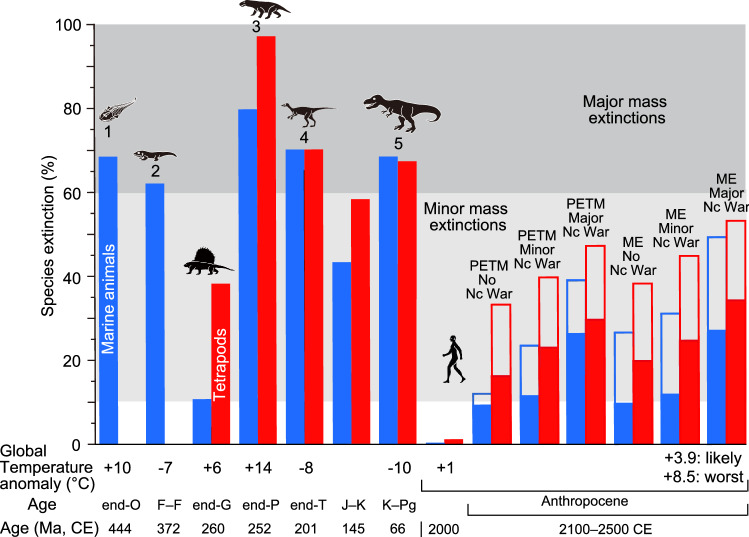
Marine animal and terrestrial tetrapod species extinction percentages corresponding to major and selected minor mass extinctions in the Phanerozoic with coincident global surface temperature anomalies. Numbers 1 to 5: the five major mass extinctions. Marine species extinction values in geologic ages shown by blue columns based on Bambach^20^. Terrestrial tetrapod species extinction values in geologic ages shown by red columns based on Benton et al.^32^ and Sahney and Benton^21^. These extinction % data are comparable because of the usage of similar methods (conventional method and substage or substage-like short intervals)^4^. Closed bars in the Anthropocene show species extinction rate (%) in the case of the most likely case. Closed plus open bars in the Anthropocene show that in the case of the worst case. Global temperature anomaly: Global surface temperature anomaly. O: Ordovician. F–F: Frasnian–Famennian boundary. G: Guadalupian. P: Permian. T: Triassic. J–K: Jurassic–Cretaceous boundary. K–Pg: Cretaceous–Paleogene boundary. Nc war: major nuclear war case. PETM: PETM case. ME: Mass extinction case. likely: most likely case. worst: worst case. Each silhouette shows a representative vertebrate animal from each age.

When nuclear war does not occur, 12–14% marine and terrestrial species loss in average is predicted under the most likely case (PETM case—mass extinction case) while a magnitude of 23–33% is predicted under the worst case (Fig. 5, Supplementary Table S9). When a major nuclear war occurs, the extinction magnitudes increase double to 28–31% species loss under the most likely case and 43–51% under the worst case (Fig. 5, Supplementary Table S9).

A minor mass extinction, 20–50% animal species loss, will occur under both the most likely and worst cases when minor or major nuclear wars and/or the worst case for global warming, pollution, and deforestation occurred. Under most likely case without minor and major nuclear wars, a mass extinction will occur, but low, 10–15% animal species loss (Fig. 5, Supplementary Table S9). The magnitude of this extinction event under the former cases will be similar to those of the end-Guadalupian (middle Permian) and Jurassic–Cretaceous (J–K) boundary crises (Fig. 5).

The most likely case corresponds to decreases in GHG emissions, pollution, and deforestation over 40–50 years corresponding to global warming total of 3.0 °C in 2100 CE and 3.8 °C in 2500 CE since 1861–1880 CE, resulting in 8% marine, 16–20% terrestrial animal species extinctions in average, and 12–14% in average of the both without any future nuclear war (the range is due to the PETM case or the mass extinction case), which less than the total extinction magnitude of the end-Guadalupian (25%). However, minor and major nuclear wars under the most likely case will cause 17–19% and 28–31% species extinctions in average, respectively. To avoid a mass extinction (> 10% animal species loss), humans must decrease GHG emissions over 20–30 years corresponding to a global warming total of less than 2.5 °C in 2100 CE since 1861–1880 CE, corresponding to less than the mid of Representative Concentration Pathway 2.6 and 4.5 (RCP 2.6 and RCP 4.5); this scenario involves decreasing pollution and deforestation at a similar rate in this century and preventing nuclear war in the future.

Humans are inducing deforestation^12,13,28^ and air/water/soil pollution due to industrial chemical reactions and the combustion of fossil fuels^3,26^, resulting in global warming^3,8,9^. At the same time, humans are facing the danger of nuclear war. The magnitudes of future extinctions mainly depend on the feasibility of (i) the CO_2_ reductions implemented by current world politics, (ii) stopping pollution, (iii) stopping deforestation, and (iv) preventing nuclear war. Humans should continuously monitor GHG emission amounts, temperature anomalies, environmental conditions including air, water, and vegetation conditions, and animal biomasses and species losses on land–near-shore sea areas; in addition, humans should conserve the environment and wildlife and prevent nuclear war to avoid a higher-magnitude crisis and to maintain ecological balance in the current human age, the Anthropocene. These measures will benefit not only animals other than humans but also humans themselves because failure to stop the four causes described above will lead to a significant decrease in biodiversity, the collapse of ecological balance, and a significant decrease in the human population.

## Methods

### Selection of environmental-biotic events to be studied

In global warming events associated with mass extinctions, the current environmental changes are similar to those recorded during the end-Ordovician, end-Guadalupian, and end-Permian mass extinctions. Therefore, I analyzed global surface temperature anomalies, mercury pollution concentrations, and deforestation percentages in these three mass extinctions and in the current crisis. The asteroid impact at the K–Pg boundary and nuclear war cause the formation of stratospheric soot aerosols distributed globally, thus inducing sunlight reductions and global cooling (impact winter and nuclear winter). I also analyzed stratospheric soot aerosols as a possible cause of future extinctions.

### Most likely case and worst case

The most likely case corresponds to the reduction of CO_2_ emissions resulting from human conduct, the protection of forests, and the introduction of anti-pollution measures in the future under the Paris Agreement on Climate change and Sustainable Development Goals (SDGs). The worst case corresponds to the scenario in which humans fail to stop increasing global surface temperatures, pollution, and deforestation until 2100–2200 CE.

I use the average of the RCP4.5 and RCP6.0 cases in the Intergovernmental Panel on Climate Change (IPCC)^8^ as the most likely case of GHG emissions, representing the middle of the four potential GHG emissions cases (RCP2.6, 4.5, 6.0, and 8.5) in Fifth Assessment Report of the IPCC^8^, approximately corresponding to the middle of SSP2-4.5 and SSP3-7.0 in Sixth Assessment Report of the IPCC^9^. The timing of decreased global GHG emissions is 2060–2080 CE. Therefore, I use the average GHG emissions and global surface temperature anomalies of the RCP4.5 and RCP6.0 cases as the most likely values and those of the RCP8.5 case as the worst-case scenario, marked by stopping GHG emissions from 2090 to 2100 CE^8,9^, as this case corresponds to the highest GHG emissions^8,9^.

### Surface temperature anomaly, environment, and extinction magnitude data

Data on surface temperature anomalies and extinction percentages are from Kaiho^4^. Changes in industrial GHG emissions and global surface temperature anomalies are sourced from the Fifth and Sixth Assessment Report of the IPCC^8,9^.

Pollution can be represented by mercury concentrations measured in sedimentary rocks recording mass extinctions^8^ and in recent sediments deposited in seas and lakes^25,26^ because mercury is toxic to plants and animals and because its sources include volcanic eruptions, meteorite impacts, and the combustion of fossil fuels^10,33^, which are common sources of pollutants, and because it can be commonly measured from sedimentary rocks recording mass extinctions^33^. The mercury concentration is related to the CO_2_ emission amount during global warming because of the common sources of mercury and CO_2_ (volcanism and fossil fuel combustion influencing global warming). Thus, the future mercury concentrations are estimated based on the CO_2_ emission amounts estimated by the IPCC^8,9^. Since mercury and the other pollutants mainly come from oil, coal, and vegetation^33^, the amount of mercury released should change in parallel with industrial CO_2_ emissions because there is a good correlation between mercury and CO_2_ emissions^11^.

Deforestation occurs by the expansion of agricultural areas and urban areas, which are strongly related to human populations^13,28^. Thus, future deforestation percentages are estimated based on estimated future population data^27^ (Supplementary Table S2). The severity of deforestation in each event is expressed by the occupancy % of the deforested area in the pre-event forest area in (i) the Permian–Triassic transition marked by the largest mass extinction based on plant fossil records^24^ and (ii) 2005–2015 CE as a representative of the Anthropocene epoch^12,13,28^ based on the actual forest area relative to the pre-agriculture phase before 4000 BP. Deforestation is related to the human population because agriculture and urbanization have caused deforestation^13,28^. I estimate the past and future deforestation percentage using human population data in the past and future^21^ based on the parallel growth of the human population and deforestation^13,28^.

Amount of stratospheric soot was calculated using a method of Kaiho and Oshima^34^ (Supplementary Table S1). I obtained global surface temperature anomaly caused by stratospheric soot using Fig. 5 of Kaiho and Oshima^34^.

I then use those data to estimate the future extinction magnitude based on the assumption that the Earth and contemporary life at the time of each crisis are more or less mutually comparable throughout time and to the present day.

I estimate the magnitude of the species animal extinction crisis between 2000 and 2500 CE using Figs. 1, 2 and Supplementary Tables S1 and S2 in each cause under the most likely case and worst case under three nuclear war scenarios (zero, minor, and major; Fig. 2d)^15^ in the PETM and mass extinction cases, respectively (Supplementary Tables S3, S4; Fig. 3). Finally, I estimate the magnitude of current animal extinction crisis by the four causes as an average of the species extinction magnitude by the four causes in Fig. 3. I use two different contribution rates of temperature anomalies, pollution, deforestation, and stratospheric soot by nuclear wars, 1:0.2:0.1:1 for marine animals and 1:0.5:1:1 for terrestrial tetrapods (different contribution case considering lower influence of pollution and deforestation to marine animals rather than terrestrial animals) and 1:1:1:1 for marine animals and 1:1:1:1 for terrestrial tetrapods (equal contribution case considering high influence of pollution and deforestation to marine animals via rain and soil erosion) (Supplementary Tables S5–S9). These contribution rates are estimated as end-members to show ranges of animal species extinction magnitude (%).

## Supplementary Information

Supplementary Information.

## Data Availability

The datasets generated and/or analysed during the current study are available in the Supplementary Information.
