# Supplementary material for: Extinction magnitude of animals in the near future
Source: Sci Rep. 2022 Nov 23;12:19593. doi: 10.1038/s41598-022-23369-5 (PMC9684554; doi:10.1038/s41598-022-23369-5)
Supplement: Supplementary file 1 — Supplementary Information. [file 41598_2022_23369_MOESM1_ESM.docx]

**Supplementary information for**

**Extinction magnitude of animals in the near future**

Kunio Kaiho

**This PDF file includes five supplementary tables (Supplementary Tables S1–S9) and references.**

**Supplementary Table S1. Data of the proxies representing the four causes of animal extinctions in the past and present**

| Age | Age | Marine | Tetrapod | Global surface | Mercury | Deforestation | Stratospheric | Impact |
| --- | --- | --- | --- | --- | --- | --- | --- | --- |
|  | (Ma) | species | species | temperature anomaly | rate |  | soot (BC) | crater |
|  |  | extinction (%) | extinction (%) | (°C) |  | (%) | (Tg) |  |
| 2000 CE | 0 | 0.2 | 1 | 1 | 3.5 | 40 |  |  |
| 1700 CE | 0 | 0 | 0.1 | 0 | 1.0 | – |  |  |
| 4000 BC | 0 | – | – | – | – | 0 |  |  |
| Late Eocene | 35.7 | 10* | 20* | – | – | – | 50 | Popigai |
| PETM | 56 | 0–10 | 0–10 | 6.5 | 3.0 | 0 |  |  |
| K–Pg | 66 | 68 | 58 | -10 | – | – | 350 | Chicxulub |
| J–K | 145 | 40 | 58 | – | – | – | 150 | Morokweng |
| End-P | 251.9 | 80 | 97 | 14 | 19.7 | 100 |  |  |
| End-G | 259.8 | 11 | 38 | 6 | 4.6 | – |  |  |
| End-O | 445–444 | 68 | – | 10 | 6.0 | – |  |  |

These data correspond to Figure 1. Data sources are the same as Figure 1. Species extinction (%) data are taken from Kaiho^4^ for geologic age crises and Barnosky et al.^1^ and Ceballos et al.^2^ for the Holocene–Anthropocene. Those for the late Eocene were estimated from Poag^35^ for marine animals and family data of Sahney and Benton^36^ for terrestrial tetrapods using the conversion relationship curve of Kaiho^4^. *Maximum values. Amount of stratospheric soot was calculated using a method of Kaiho and Oshima^34^ and sedimentary rock and the thickness data of Koeberl and Reimold^37^ for Morokweng and those of Masaitis et al.^38^ for Popigai.

**Supplementary Table S2. Changes in the proxies representing the four causes of animal extinctions from 1700–2500 CE**

|  | Temp | Temp | CO_2_ | CO_2_ emission | Pollution | Pollution max | Human | Human | Deforestation | Deforestation | Nc Soot | Nc Soot | Nc Soot |
| --- | --- | --- | --- | --- | --- | --- | --- | --- | --- | --- | --- | --- | --- |
| CE |  | Max | emission | maximum | Mercury | Mercury | population | population | from 4000 BC | from 4000 BC | no case | minor case | major case |
|  |  |  | (Gt/y) | (Gt/y) | rate | rate max | (Billion) | max (Billion) | (%) | Maximum (%) | (Tg) | (Tg) | (Tg) |
| -4000 | – | – | – | – | – | – | 0.01 | 0.01 | 0 | 0 | – | – | – |
| 1700 | 0 | 0 | 0 | 0 | 1 | 1 | 0.6 | 0.6 | 4 | 4 | 0 | 0 | 0 |
| 1800 | 0 | 0 | 0 | 0 | 1.0 | 1.0 | 0.99 | 0.99 | 6.6 | 6.6 | 0 | 0 | 0 |
| 1900 | 0 | 0 | 4 | 4 | 1.3 | 1.3 | 1.67 | 1.67 | 11 | 11 | 0 | 0 | 0 |
| 2000 | 1 | 1 | 40 | 40 | 3.5 | 3.5 | 6 | 6 | 40 | 40 | 0 | 50 | 150 |
| 2100 | 3 | 4.5 | 35 | 100 | 3.6 | 7.7 | 10.9 | 14 | 73 | 80 | 0 | 50 | 150 |
| 2200 | 3.5 | 7.5 | 3 | 20 | 1.6 | 2 | 10 | 13 | 67 | 80 | 0 | 50 | 150 |
| 2300 | 3.7 | 8 | 2 | 6 | 1.5 | 1.8 | 9.6 | 12 | 63 | 80 | 0 | 50 | 150 |
| 2400 | 3.8 | 8.3 | 1.5 | 6 | 1.5 | 1.7 | 9.3 | 12 | 61 | 80 | 0 | 50 | 150 |
| 2500 | 3.9 | 8.5 | 1 | 7 | 1.4 | 1.6 | 9 | 12 | 60 | 80 | 0 | 50 | 150 |

These data correspond to Figure 2. Data sources are the same as Figure 2. CE: calendar year Temp: temperature anomaly (°C). max: maximum. no case: no occurrence case. Mercury rate values in future are calculated from CO_2_ emission in this table based on CO_2_ data of IPCC^8,9^ and O’Neill et al.^39^. Population data after Roser^27^ and Basten et al.^40^. Future deforestation data are calculated from the population data in this table.

**Supplementary Table S3. Changes in the animal extinction magnitudes (%) by global warming from 1700–2500 CE (PETM case for temperature)**

|  | Temp | Temp | Temp | Temp |
| --- | --- | --- | --- | --- |
| CE | Marine | Marine | Terrestrial | Terrestrial |
|  | likely | worst | likely | worst |
| 1700 | 0 | 0 | 0.2 | 0.2 |
| 1800 | 0 | 0 | 0.25 | 0.25 |
| 1900 | 0 | 0 | 0.6 | 0.6 |
| 2000 | 0.1 | 0.1 | 1 | 1 |
| 2100 | 2 | 4 | 4 | 8 |
| 2200 | 2 | 5 | 4 | 9 |
| 2300 | 3 | 6 | 5 | 10 |
| 2400 | 3 | 6 | 5 | 10 |
| 2500 | 3 | 6 | 5 | 10 |
| Max | 3 | 6 | 5 | 10 |

These data correspond to Figure 3a. Max: Maximum, which represent the final extinction percentages. These data are based on no mass extinction at the PETM^26,28^. No mass extinction is defined by <10% species extinction in this study. This table is based on similarity between the PETM and ongoing crisis in global warming. Temp: temperature anomaly (°C). likely: most likely case. worst: worst case. Max: maximum value.

**Supplementary Table S4. Changes in the animal extinction magnitudes (%) by each cause from 1700–2500 CE (mass extinction case for temperature)**

|  | Temp | Temp | Temp | Temp | Pollution | Pollution | Pollution | Pollution | Deforest | Deforest | Deforest | Deforest | Nc war | Nc war | Nc war | Nc war |
| --- | --- | --- | --- | --- | --- | --- | --- | --- | --- | --- | --- | --- | --- | --- | --- | --- |
| CE | Marine | Marine | Terrestrial | Terrestrial | Marine | Marine | Terrestrial | Terrestrial | Marine | Marine | Terrestrial | Terrestrial | Marine | Marine | Terrestrial | Terrestrial |
|  | likely | worst | likely | worst | likely | worst | likely | worst | likely | worst | likely | worst | likely | worst | likely | worst |
| 1700 | 0 | 0 | 0.2 | 0.2 | 0 | 0 | 0.2 | 0.2 | 0 | 0 | 0.2 | 0.2 | 0 | 0 | 0 | 0 |
| 1800 | 0 | 0 | 0.25 | 0.25 | 0 | 0 | 0.25 | 0.25 | 0 | 0 | 0.25 | 0.25 | 0 | 0 | 0 | 0 |
| 1900 | 0 | 0 | 0.6 | 0.6 | 0 | 0 | 0.6 | 0.6 | 0 | 0 | 0.6 | 0.6 | 0 | 0 | 0 | 0 |
| 2000 | 0.1 | 0.1 | 1 | 1 | 0.1 | 0.1 | 1 | 1 | 0.1 | 0.1 | 1 | 1 | 0 | 0 | 0 | 0 |
| 2100 | 3 | 7 | 17 | 27 | 1.1 | 60 | 4.8 | 70 | 45 | 53 | 53 | 65 | 10 | 55 | 25 | 55 |
| 2200 | 4 | 27 | 20 | 50 | 0 | 0 | 0.43 | 0.55 | 37 | 53 | 48 | 65 | 10 | 55 | 25 | 55 |
| 2300 | 5 | 40 | 21 | 54 | 0 | 0 | 0.4 | 0.49 | 32 | 53 | 38 | 65 | 10 | 55 | 25 | 55 |
| 2400 | 6 | 45 | 22 | 56 | 0 | 0 | 0.38 | 0.46 | 28 | 53 | 33 | 65 | 10 | 55 | 25 | 55 |
| 2500 | 7 | 49 | 23 | 58 | 0 | 0 | 0.37 | 0.43 | 27 | 53 | 32 | 65 | 10 | 55 | 25 | 55 |
| Max | 7 | 49 | 23 | 58 | 1.1 | 60 | 5 | 70 | 45 | 53 | 53 | 65 | 10 | 55 | 25 | 55 |

These data correspond to Figure 3b–3e. Max: Maximum, which represent the final extinction percentages. Temp: temperature anomaly (°C). likely: most likely case. worst: worst case. Max: maximum value.

**Supplementary Table S5. Changes in the animal extinction magnitudes (%) by four causes from 1700–2500 CE (PETM case and different contribution case)**

| Contribution | 1:0.2:0.1:1 | 1:0.5:1:1 | 1:0.2:0.1:1 | 1:0.5:1:1 | 1:0.2:0.1:1 | 1:0.5:1:1 | 1:0.2:0.1:1 | 1:0.5:1:1 | 1:0.2:0.1:1 | 1:0.5:1:1 | 1:0.2:0.1:1 | 1:0.5:1:1 |
| --- | --- | --- | --- | --- | --- | --- | --- | --- | --- | --- | --- | --- |
| Nc W level | No Nc W | No Nc W | No Nc W | No Nc W | Minor Nc W | Minor Nc W | Minor Nc W | Minor Nc W | Major Nc W | Major Nc W | Major Nc W | Major Nc W |
| Case | likely | likely | worst | worst | likely | likely | worst | worst | likely | likely | worst | worst |
| CE / Region | Marine | Terrestrial | Marine | Terrestrial | Marine | Terrestrial | Marine | Terrestrial | Marine | Terrestrial | Marine | Terrestrial |
| 1700 | 0 | 0.14 | 0 | 0.14 | 0 | 0.14 | 0 | 0.14 | 0 | 0.13 | 0 | 0.14 |
| 1800 | 0 | 0.18 | 0 | 0.18 | 0 | 0.18 | 0 | 0.18 | 0 | 0.16 | 0 | 0.18 |
| 1900 | 0 | 0.43 | 0 | 0.43 | 0 | 0.43 | 0 | 0.43 | 0 | 0.38 | 0 | 0.43 |
| 2000 | 0.06 | 0.71 | 0.06 | 0.71 | 0.06 | 0.71 | 0.06 | 0.71 | 0.06 | 0.63 | 0.06 | 0.71 |
| 2100 | 2.92 | 16.97 | 9.26 | 30.86 | 8.14 | 24.11 | 14.91 | 37.71 | 27.7 | 28.6 | 34.48 | 46.29 |
| 2200 | 2.48 | 14.92 | 4.48 | 21.22 | 7.7 | 22.06 | 11 | 28.65 | 27.26 | 26.8 | 30.57 | 37.22 |
| 2300 | 2.7 | 12.34 | 4.91 | 21.5 | 7.91 | 19.49 | 11 | 28.64 | 27.48 | 24.55 | 30.57 | 37.21 |
| 2400 | 2.52 | 10.91 | 4.91 | 21.49 | 7.74 | 18.05 | 11 | 28.64 | 27.3 | 23.3 | 30.57 | 37.21 |
| 2500 | 2.48 | 10.62 | 4.91 | 21.49 | 7.7 | 17.77 | 11 | 28.63 | 27.26 | 23.05 | 30.57 | 37.2 |
| Max Ext | 3 | 17 | 9 | 31 | 8 | 24 | 15 | 38 | 28 | 29 | 34 | 46 |

These data correspond to Figure 3e–3f. Max Ext: Maximum Extinction. These values represent the final extinction percentages in Figure 5. Contribution: Contribution rate of each cause (global surface temperature anomaly, pollution, deforestation, and sunlight reduction). See Methods on the calculation for those extinction percentages. No Nc W: no nuclear war case. Minor Nc W: minor nuclear war case. Major Nc W: major nuclear war case. likely: most likely case. worst: worst case. Max Ext: maximum extinction rate value (%).

**Supplementary Table S6. Changes in the animal extinction magnitudes by four causes from 1700–2500 CE (PETM case and equal contribution case)**

| Contribution | 1:1:1:1 | 1:1:1:1 | 1:1:1:1 | 1:1:1:1 | 1:1:1:1 | 1:1:1:1 | 1:1:1:1 | 1:1:1:1 | 1:1:1:1 | 1:1:1:1 | 1:1:1:1 | 1:1:1:1 |
| --- | --- | --- | --- | --- | --- | --- | --- | --- | --- | --- | --- | --- |
| Nc W level | No Nc W | No Nc W | No Nc W | No Nc W | Minor Nc W | Minor Nc W | Minor Nc W | Minor Nc W | Major Nc W | Major Nc W | Major Nc W | Major Nc W |
| Case | likely | likely | worst | worst | likely | likely | worst | worst | likely | likely | worst | worst |
| CE / Region | Marine | Terrestrial | Marine | Terrestrial | Marine | Terrestrial | Marine | Terrestrial | Marine | Terrestrial | Marine | Terrestrial |
| 1700 | 0.00 | 0.15 | 0.00 | 0.15 | 0 | 0.14 | 0 | 0.14 | 0 | 0.15 | 0 | 0.15 |
| 1800 | 0.00 | 0.19 | 0.00 | 0.19 | 0 | 0.18 | 0 | 0.18 | 0 | 0.19 | 0 | 0.19 |
| 1900 | 0.00 | 0.45 | 0.00 | 0.45 | 0 | 0.43 | 0 | 0.43 | 0 | 0.45 | 0 | 0.45 |
| 2000 | 0.08 | 0.75 | 0.08 | 0.75 | 0.06 | 0.71 | 0.02 | 0.71 | 0.08 | 0.75 | 0.05 | 0.75 |
| 2100 | 12.53 | 15.45 | 15.00 | 35.50 | 15.03 | 21.7 | 32.5 | 41.75 | 26.28 | 29.2 | 43.75 | 49.25 |
| 2200 | 10.25 | 13.11 | 15.75 | 18.89 | 12.75 | 19.36 | 18.25 | 25.14 | 24 | 26.86 | 29.5 | 32.64 |
| 2300 | 9.25 | 10.85 | 15.75 | 18.87 | 11.75 | 17.1 | 18.25 | 25.12 | 23 | 24.6 | 29.5 | 32.62 |
| 2400 | 8.25 | 9.60 | 15.75 | 18.87 | 10.75 | 15.85 | 18.25 | 25.12 | 22 | 23.35 | 29.5 | 32.62 |
| 2500 | 8.00 | 9.34 | 15.75 | 18.86 | 10.5 | 15.59 | 18.25 | 25.11 | 21.75 | 23.09 | 29.5 | 32.61 |
| Max Ext | 13 | 16 | 16 | 36 | 15 | 22 | 33 | 42 | 26 | 29 | 44 | 49 |

These data correspond to Figure 3e–3f. Max Ext: Maximum Extinction. These values represent the final extinction percentages in Figure 5. Contribution: Contribution rate of each cause (global surface temperature anomaly, pollution, deforestation, and sunlight reduction). See Methods on the calculation for those extinction percentages. No Nc W: no nuclear war case. Minor Nc W: minor nuclear war case. Major Nc W: major nuclear war case. likely: most likely case. worst: worst case.

**Supplementary Table S7. Changes in the animal extinction magnitudes by four causes from 1700–2500 CE (mass extinction case and different contribution case)**

| Contribution | 1:0.2:0.1:1 | 1:0.5:1:1 | 1:0.2:0.1:1 | 1:0.5:1:1 | 1:0.2:0.1:1 | 1:0.5:1:1 | 1:0.2:0.1:1 | 1:0.5:1:1 | 1:0.2:0.1:1 | 1:0.5:1:1 | 1:0.2:0.1:1 | 1:0.5:1:1 |
| --- | --- | --- | --- | --- | --- | --- | --- | --- | --- | --- | --- | --- |
| Nc W level | No Nc W | No Nc W | No Nc W | No Nc W | Minor Nc W | Minor Nc W | Minor Nc W | Minor Nc W | Major Nc W | Major Nc W | Major Nc W | Major Nc W |
| Case | likely | likely | worst | worst | likely | likely | worst | worst | likely | likely | worst | worst |
| CE / Region | Marine | Terrestrial | Marine | Terrestrial | Marine | Terrestrial | Marine | Terrestrial | Marine | Terrestrial | Marine | Terrestrial |
| 1700 | 0.00 | 0.14 | 0.00 | 0.14 | 0.00 | 0.14 | 0.00 | 0.14 | 0.00 | 0.14 | 0.00 | 0.14 |
| 1800 | 0.00 | 0.18 | 0.00 | 0.18 | 0.00 | 0.18 | 0.00 | 0.18 | 0.00 | 0.18 | 0.00 | 0.18 |
| 1900 | 0.00 | 0.43 | 0.00 | 0.43 | 0.00 | 0.43 | 0.00 | 0.43 | 0.00 | 0.43 | 0.00 | 0.43 |
| 2000 | 0.06 | 0.71 | 0.06 | 0.71 | 0.06 | 0.71 | 0.06 | 0.71 | 0.06 | 0.71 | 0.04 | 0.71 |
| 2100 | 3.36 | 20.69 | 10.57 | 36.29 | 7.70 | 27.83 | 14.91 | 43.43 | 27.27 | 36.40 | 54.61 | 52.00 |
| 2200 | 3.35 | 19.49 | 14.04 | 32.94 | 7.70 | 26.63 | 18.39 | 40.08 | 27.26 | 35.20 | 35.65 | 48.65 |
| 2300 | 3.57 | 16.91 | 19.70 | 34.07 | 7.91 | 24.06 | 24.04 | 41.21 | 27.48 | 32.63 | 41.30 | 49.78 |
| 2400 | 3.83 | 15.77 | 21.87 | 34.64 | 8.17 | 22.91 | 26.22 | 41.78 | 27.74 | 31.48 | 43.48 | 50.35 |
| 2500 | 4.22 | 15.77 | 23.61 | 35.20 | 8.57 | 22.91 | 27.96 | 42.35 | 28.13 | 31.48 | 45.22 | 50.92 |
| Max Ext | 4 | 21 | 24 | 36 | 9 | 28 | 28 | 43 | 28 | 36 | 55 | 52 |

These data correspond to Figure 3e–3f. Max Ext: Maximum Extinction, which values represent the final extinction percentages in Figure 5. Contribution: Contribution rate of each cause (global surface temperature anomaly, pollution, deforestation, and sunlight reduction). See Methods on the calculation for those extinction percentages. No Nc W: no nuclear war case. Minor Nc W: minor nuclear war case. Major Nc W: major nuclear war case. likely: most likely case. worst: worst case.

**Supplementary Table S8. Changes in the animal extinction magnitudes by four causes from 1700–2500 CE (mass extinction case and equal contribution case)**

| Contribution | 1:1:1:1 | 1:1:1:1 | 1:1:1:1 | 1:1:1:1 | 1:1:1:1 | 1:1:1:1 | 1:1:1:1 | 1:1:1:1 | 1:1:1:1 | 1:1:1:1 | 1:1:1:1 | 1:1:1:1 |
| --- | --- | --- | --- | --- | --- | --- | --- | --- | --- | --- | --- | --- |
| Nc W level | No Nc W | No Nc W | No Nc W | No Nc W | Minor Nc W | Minor Nc W | Minor Nc W | Minor Nc W | Major Nc W | Major Nc W | Major Nc W | Major Nc W |
| Case | likely | likely | worst | worst | likely | likely | likely | likely | likely | likely | worst | worst |
| CE / Region | Marine | Terrestrial | Marine | Terrestrial | Marine | Terrestrial | Marine | Terrestrial | Marine | Terrestrial | Marine | Terrestrial |
| 1700 | 0.00 | 0.15 | 0.00 | 0.15 | 0.00 | 0.15 | 0.00 | 0.15 | 0.00 | 0.15 | 0.00 | 0.15 |
| 1800 | 0.00 | 0.19 | 0.00 | 0.19 | 0.00 | 0.19 | 0.00 | 0.19 | 0.00 | 0.19 | 0.00 | 0.19 |
| 1900 | 0.00 | 0.45 | 0.00 | 0.45 | 0.00 | 0.45 | 0.00 | 0.45 | 0.00 | 0.45 | 0.00 | 0.45 |
| 2000 | 0.08 | 0.75 | 0.08 | 0.75 | 0.08 | 0.75 | 0.08 | 0.75 | 0.08 | 0.75 | 0.08 | 0.75 |
| 2100 | 12.28 | 18.70 | 30.00 | 40.50 | 14.78 | 24.95 | 32.5 | 46.75 | 26.03 | 32.45 | 43.75 | 54.25 |
| 2200 | 10.25 | 17.11 | 20.00 | 28.89 | 12.75 | 23.36 | 22.5 | 35.14 | 24.00 | 30.86 | 33.75 | 42.64 |
| 2300 | 9.25 | 14.85 | 23.25 | 29.87 | 11.75 | 21.10 | 25.75 | 36.12 | 23.00 | 28.60 | 37.00 | 43.62 |
| 2400 | 8.50 | 13.85 | 24.50 | 30.37 | 11.00 | 20.10 | 27 | 36.62 | 22.25 | 27.60 | 38.25 | 44.12 |
| 2500 | 8.50 | 13.84 | 25.50 | 30.86 | 11.00 | 20.09 | 28 | 37.11 | 22.25 | 27.59 | 39.25 | 44.61 |
| Max Ext | 12 | 19 | 30 | 41 | 15 | 25 | 33 | 47 | 26 | 32 | 44 | 54 |

These data correspond to Figure 3e–3f. Max Ext: Maximum Extinction. These values represent the final extinction percentages in Figure 5. Contribution: Contribution rate of each cause (global surface temperature anomaly, pollution, deforestation, and sunlight reduction). See Methods on the calculation for those extinction percentages. No Nc W: no nuclear war case. Minor Nc W: minor nuclear war case. Major Nc W: major nuclear war case. likely: most likely case. worst: worst case.

**Supplementary Table S9. Animal extinction magnitudes till 2500 CE**

| PETM or |  |  |  |  |  |  |  | Marine and |
| --- | --- | --- | --- | --- | --- | --- | --- | --- |
| mass | Region | Marine | | Terrestrial | | Marine | Terrestrial | terrestrial |
| extinction (ME) | Case / contribution | 1:0.2:0.1:1 | 1:1:1:1 | 1:0.5:1:1 | 1:1:1:1 | average | average | average |
| PETM | No nuclear war likely case | 3 | 13 | 17 | 15 | 8 | 16 | 12 |
| PETM | No nuclear war worst case | 9 | 16 | 31 | 36 | 12.5 | 33.5 | 23 |
| PETM | Minor nuclear war likely case | 8 | 15 | 24 | 22 | 11.5 | 23 | 17 |
| PETM | Minor nuclear war worst case | 15 | 33 | 38 | 42 | 24 | 40 | 32 |
| PETM | Major nuclear war likely case | 28 | 26 | 29 | 29 | 27 | 29 | 28 |
| PETM | Major nuclear war worst case | 34 | 44 | 46 | 49 | 39 | 47.5 | 43 |
| ME | No nuclear war likely case | 4 | 12 | 21 | 19 | 8 | 20 | 14 |
| ME | No nuclear war worst case | 24 | 30 | 36 | 41 | 27 | 38.5 | 33 |
| ME | Minor nuclear war likely case | 9 | 15 | 28 | 25 | 12 | 26.5 | 19 |
| ME | Minor nuclear war worst case | 28 | 33 | 43 | 47 | 30.5 | 45 | 38 |
| ME | Major nuclear war likely case | 28 | 26 | 36 | 32 | 27 | 34 | 31 |
| ME | Major nuclear war worst case | 55 | 44 | 52 | 54 | 49.5 | 53 | 51 |

Animal extinction magnitudes correspond to maximum species extinction (%) in Figure 4. Most of the maximum values will appear in ~2100 CE.

**References**

35. Poag, C.W. Roadblocks on the kill curve: testing the Raup hypothesis. *Palaios* **12,** 582–590 (1997).

36. Sahney, S. & Benton, M. J. The impact of the Pull of the Recent on the fossil record of tetrapods. *Evol. Ecol. Res.* **18,** 7–23 (2017).

37. Koeberl, C. & Reimold, W. U. Geochemistry and petrography of impact breccias and target rocks from the 145 Ma Morokweng impact structure, South Africa. *Geochim. Cosmochim. Acta* **67,** 1837–1862 (2003).

38. Masaitis, V. L., Naumov, M. V. & Mashchak, M. S. Original diameter and depth of erosion of the Popigai impact crater, Russia. *Geol. Soc. Am. Spec. Pap.* **384,** 131–140 (2005).

39. O'Neill, B. C. et al. The Scenario Model Intercomparison Project (ScenarioMIP) for CMIP6. *Geosci. Model Dev*. **9,** 3461–3482 (2016).

40. Basten, S., Lutz, W. & Scherbov, S. Very long range global population scenarios to 2300 and the implications of sustained low fertility. Demographic Res. **28;** <http://dx.doi.org/10.4054/DemRes.2013.28.39> (2013).
